# The phenomenon of anticipation in fencing. An applicability approach

**DOI:** 10.3389/fspor.2024.1387013

**Published:** 2024-04-25

**Authors:** Zbigniew Borysiuk, Mariusz Konieczny, Monika Błaszczyszyn, Wiesław Błach, Zbigniew Obmiński

**Affiliations:** ^1^ Faculty of Physical Education and Physiotherapy, Opole University of Technology, Opole, Poland; ^2^ Wroclaw University of Health and Sports Sciences, Wrocław, Poland; ^3^ Institute of Sport, National Research Institute, Warsaw, Poland

**Keywords:** preparatory period, reaction time, movement time, electromyography, ground reaction forces

## Abstract

**Introduction:**

The aim of the study was to determine the structure of muscular activity and ground reaction forces during the preparatory period and the execution of a fencing lunge at the opponent's torso. The analysis focused on the correlations between three phases of a fencing technical action in the context of factors of temporal anticipation.

**Methods:**

Six female épée fencers from the Polish National Fencing Team participated in the study. The research tools included electromyography (EMG), ground reaction force (GRF) platforms, and the OptiTrack motion capture system. The fencers performed the lunge three times in response to visual cues from the coach. By integrating the testing system, the EMG signal indices of the fencers' upper and lower limbs and the vertical force values of the fencers' front and rear leg muscles were obtained simultaneously.

**Results:**

The results of the study demonstrated the key role of five muscles: BICEPS BRACHII, LAT TRICEPS, EXTCARP RAD, BICEPS FEMORIS and MED GAS in influencing the speed of lunge execution. In addition, a significant correlation was found between the EMG signal of the gastrocnemius muscle of the rear leg and the movement time (MT) phase of the lunge execution.

**Discussion:**

The anticipatory activation of the EMG signal in relation to the vertical force waveforms generated by the ground forces response platform in the 15–30 ms interval was demonstrated. Finally, the importance of the preparatory period for the effectiveness of the fencing lunge was highlighted based on the phenomenon of anticipation.

## Introduction

Anticipation of opponents' actions in sports based on open motor habits, including combat sports, involves perception and imaging of upcoming situations related to the selection of appropriate technical-tactical actions (1, 2). Athletes, as part of their anticipatory processes based on experience, identify tactical goals set by opponents in sports competition. The key to understanding psychomotor responses based on anticipatory processes is the sensorimotor response paradigm formulated by Czajkowski (3). An essential component of this paradigm is the preparatory period, which occurs prior to the execution of a technical action and is characterized by readiness to perform an intentional action. It includes the planning of a specific action involving mental processes, i.e., concentration and focus of attention, before the anticipated movement task. The second stage, after the occurrence of the anticipated stimulus signal, is the latency period, defined as reaction time (RT) (4). According to Schmidt (5) and Rosenbaum (6), the RT consists of three phases: identification, selection, and programming of the sensorimotor response. The RT ends with the initiation of the movement time (MT) phase, which is expressed by the activation of the muscles involved in a given motor action.

With the advancement of new technologies, primarily the use of surface electromyography and force plates, the traditional approach to the sensorimotor approach today requires some revision. According to a number of studies, muscle tension and pressure on the ground reaction force platforms occur during both the preparatory and latency phases of the sensorimotor response. There is no doubt that athletes “launch” their motor programs in anticipation, creating a predetermined structure of muscle tension in a given movement pattern (7, 8). Anticipating the opponent's intentions gives a competitor more time to prepare and execute effective responses and to make appropriate adjustments depending on changes in the technical-tactical situation (9).

It is essential to use research tools that allow precise recording of the signal to which the athletes being studied are responding, while simultaneously recording the EMG curves of the muscles being studied (10). In the present study, markers from the OptiTrack system were placed on the coach's and fencer's epee blade guards. A forward movement of the coach's hand initiated the fencer's response, which consisted of an immediate fencing lunge to a specific location on the coach's torso. Accordingly, the reaction time from “coach's marker” to “fencer's marker” was determined using the markers (shown as vertical bars in the diagrams). In addition, the movement time from the “fencer marker” to the completion of the action, i.e., the “moment of touch”, was also determined. Since the EMG curves were visible before the start of the movement and also during the movement, heuristic analyses of their course were carried out. However, for the sake of precision of the EMG signal analysis, the “muscle onset” procedure was used, as described in the subsection “Materials, tools and research procedures”. In addition, given the importance of postural adjustment, two integrated ground reaction force (GRF) plates were used to measure the timing and reaction force of the fencers' legs during the execution of the lunge.

In terms of applicability, the fencers in the study were selected because of the specifics of their training, which, apart from free fencing matches, consists mainly of individual lessons with a coach. During these training sessions, fencing coaches simulate various technical actions in combination with the expected reactions of the opponents, to which adequate responses must be applied (11). In this sense, a typical fencer's training is similar to a real sports fight, and studies involving coaches and fencers have particular applicability, especially in the context of different types of anticipation (12, 13).

To accomplish the goals of the study, elite fencers were selected because they most clearly manifest the attributes of training that perfect the complexity of anticipatory processes (14). The variety and perfect mastery of different movement patterns, enhanced by practice, allows expert fencers to reliably anticipate the actions of their opponents. According to researchers, about 80%–90% of technical and tactical actions in sports with open motor habits are anticipatory in nature.

The present study aimed to address the following research issues

- The relationships between the timing of muscle activation during the performance of a fencing lunge during reaction time (RT) (coach marker—fencer marker) and movement time (MT) (fencer marker—moment of contact);

- Relationships between timing and volume of EMG and GRF platform signals.

Furthermore, a heuristic analysis of the whole movement pattern of the fencing lunge was performed, taking into account the preparatory period.

## Methods

Six female epee fencers of the Polish Olympic Fencing Team, aged 24.6 ± 6.2 years, who have been practicing fencing for several years with international success in junior and senior age categories, were selected for the study.

Eight muscles of the fencers' upper and lower limbs were assessed using surface EMG: biceps brachii (BICEPS BR), triceps lateralis (LAT TRICEPS), flexor carpi ulnaris (FLEX CARP U), extensor carpi radialis (EXT CARP RAD), biceps femoris (BICEPS FEM), rectus femoris (RFCTUS FEM), gastrocnemius lateralis (LAT GAS), and gastrocnemius medialis (MED GAS).

Two combined Kistler force plates were used to assess ground reaction forces to classify the movement patterns of the fencers' front and rear legs. Both ground reaction force plates were synchronized with TTL signals. The following labels were used: Fz rear (rear leg vertical force) and Fz front (front leg vertical force).

**PHOTO 1:**
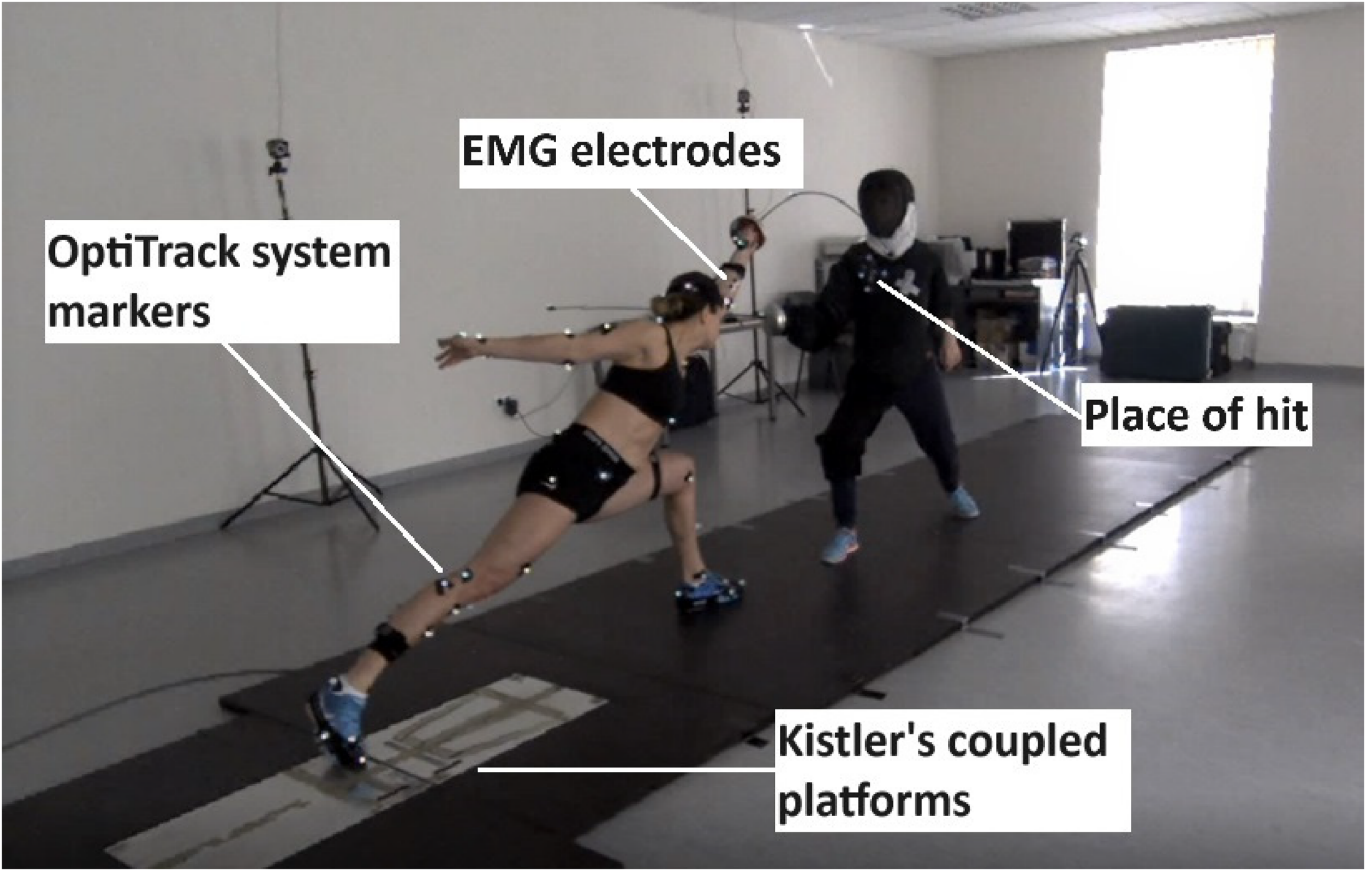
A fencer performing a lunge at the coach's torso. Written informed consent was obtained from the individual for the publication of any potentially identifiable images or data included in the article.

A motion capture system (OptiTrack, NaturalPoint, Inc., Corvallis, USA) consisting of eight cameras and markers that define a specific body position was used to record the movement sequences of the fencers and the coach. The markers were placed on the epee blades and guards of the coach and fencers and on the body of the coach: three markers on the weapon (fencer and coach) and four on the torso of the coach, marking a 10 × 10 square target area (“place of hit”) (Photo 1).

The moment of the coach's movement (coach marker) was determined in terms of muscle activity (EMG) and ground reaction forces (GRF). Then, the procedure of the initial muscle activity was adopted according to the algorithm: the mean EMG signal + 3SD calculated from 100 ms at the moment of taking the on guard stance (muscle onset), determined from the fencer's sword hand marker, and the moment of touch determined by the MATLAB automatic function findchangepts.

The signal to perform the epee thrust with a lunge at the coach's torso was initiated by the coach making a forward motion with his hand. Each fencer performed three lunge attempts, from which the best was selected.

The project was approved by the Bioethics Committee of the Medical Chamber (Resolution No. 237 of December 13, 2016) in accordance with the guidelines of the Declaration of Helsinki for the conduct of clinical trials on human subjects.

### Statistical analysis

The relationships between maximum ground reaction forces and EMG were determined using the Pearson correlation coefficient after checking the normality of the distribution with the Shapiro-Wilk test. The analysis was performed using JAMOVI software (jamovi.org).

## Results

**Figure 1:**
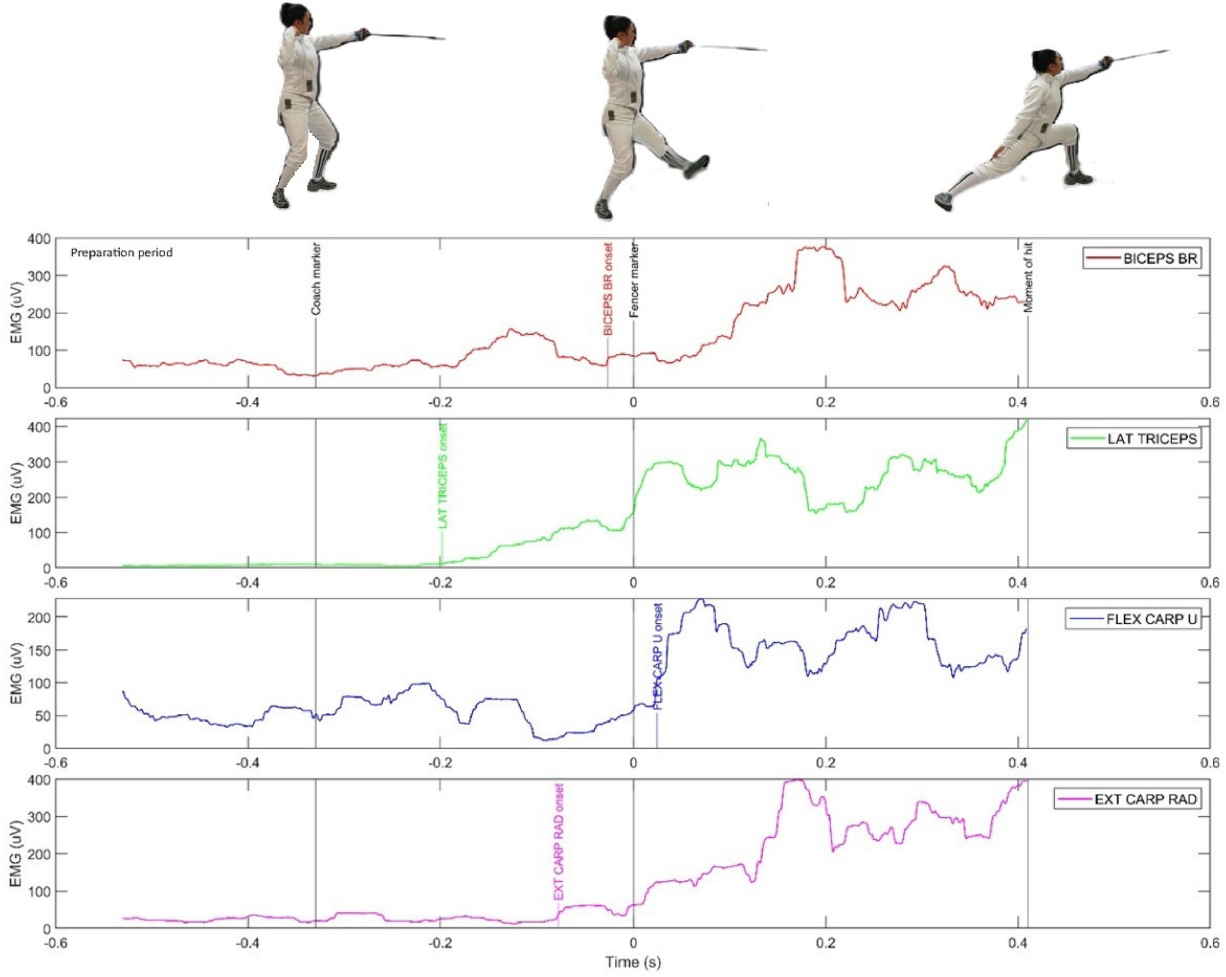
The activity of four muscles of the attacking arm in the preparatory period (in the latency phase of the sensorimotor response) during the execution of a fencing lunge.

**Figure 2:**
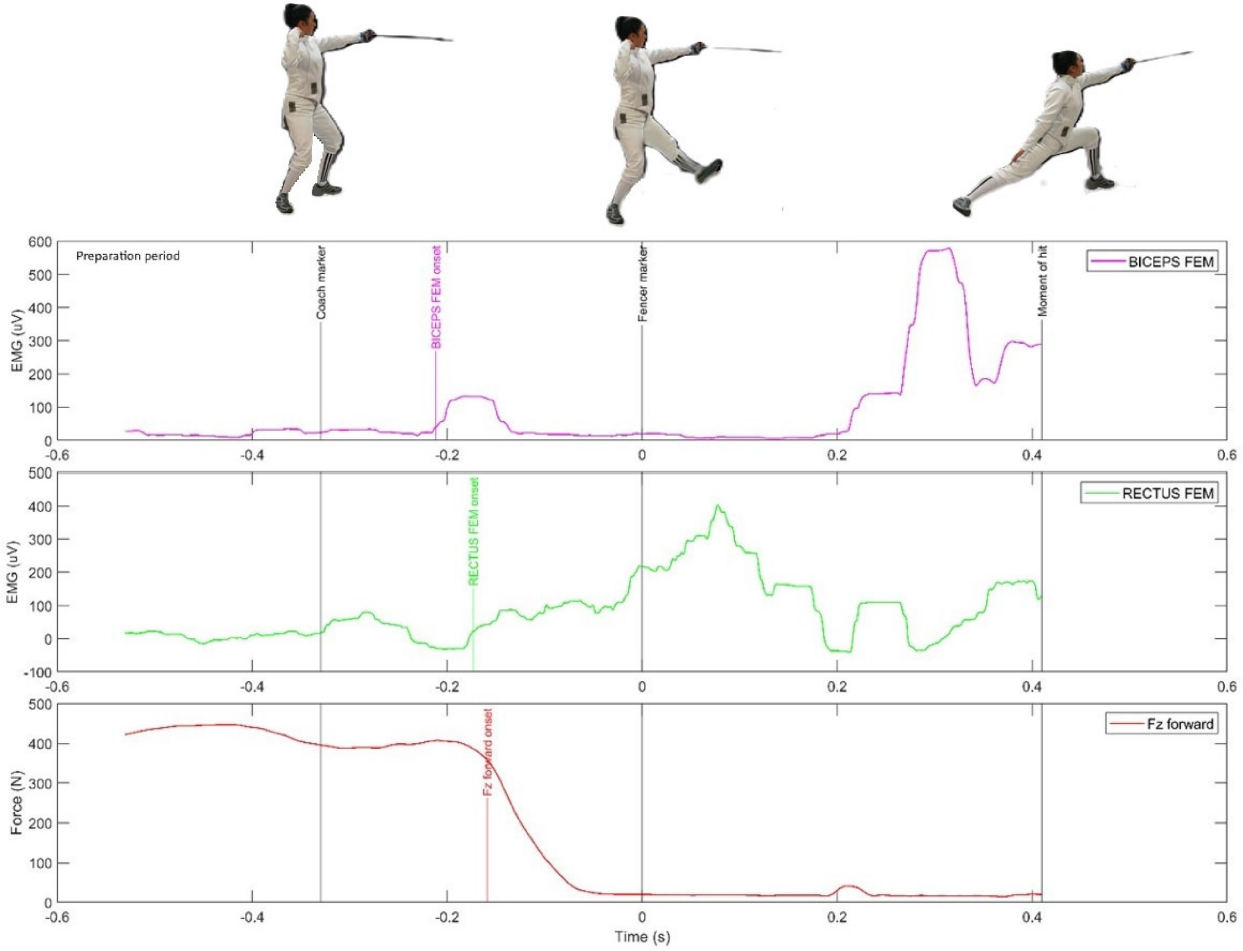
The activity of two muscles of the front leg and the vertical force in the preparatory period (in the latency phase of the sensorimotor response) during the execution of the fencing lunge.

**Figure 3:**
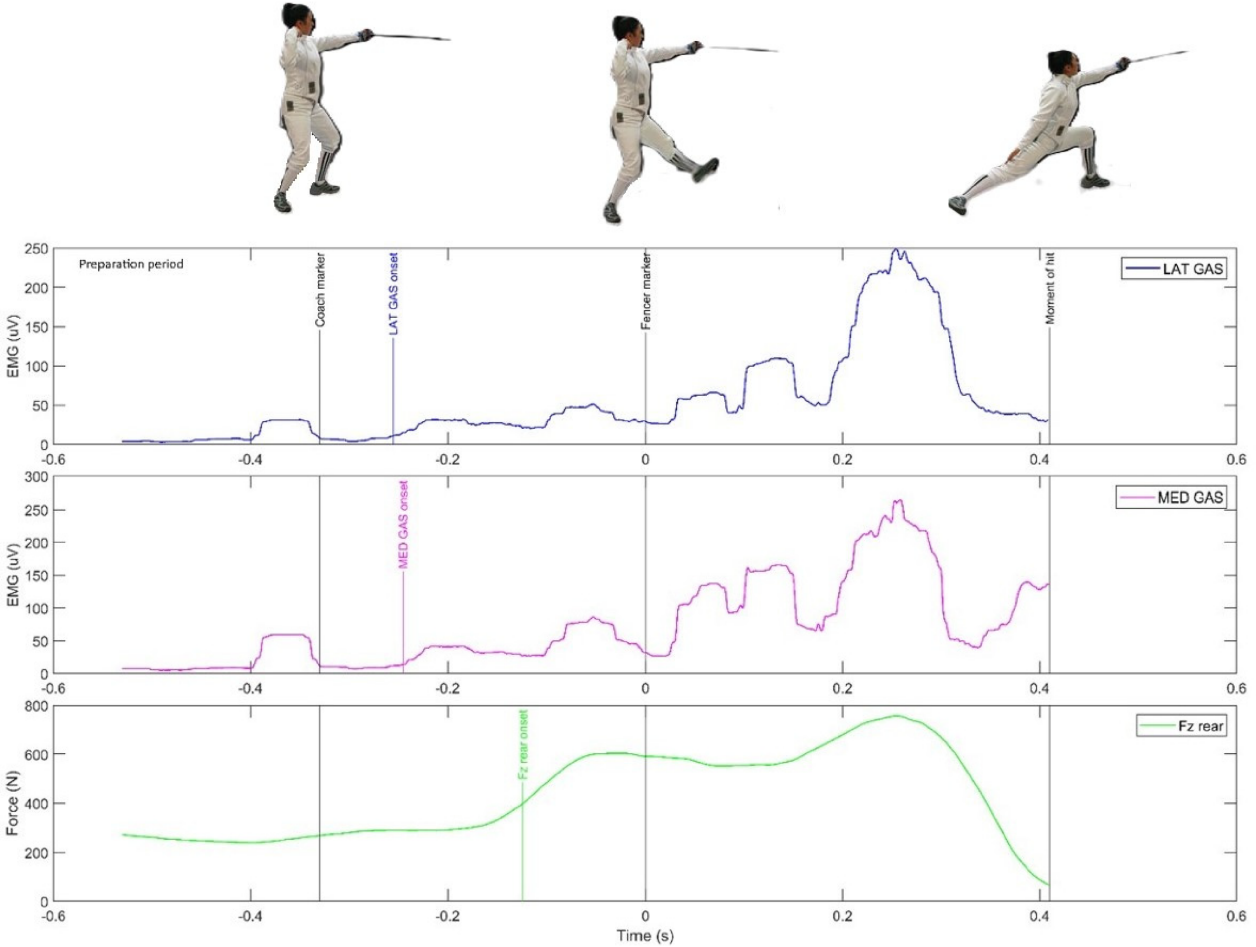
The activity of two muscles of the rear leg and the vertical force in the preparatory period (in the latency phase of the sensorimotor response) during the execution of the fencing lunge.

Analysis of the EMG and vertical force curves of the lower limbs of the six female fencers revealed close similarities in the structure of their muscle activation. Figures 1–3 show the performance of a fencer with a 31 ms delay in the vertical force of the rear leg (Fz rear) relative to the activation of the EMG signal.

Figures 1–3 show the EMG activity of the eight active muscles and the vertical force curves of the lower limbs during the execution of a fencing lunge. A coordinated activity of all the limbs of the fencers was observed, with the least role played by the non-attacking arm, which only helps to maintain balance after a hit. The primary role is attributed to the fencer's rear leg and the dominant arm that delivers the strike. As shown in Figures 2, 3, the RECTUS FEM and BICEPS FEM muscles are activated later than the rear leg muscles, but in slight synergy with the peak of the vertical force of the front leg (Fz forward). During the dynamic extension of the rear leg, the front leg is lifted off the ground and during the extension, the RECTUS FEM extensor is activated first (EMG = 400 uV). Then the BICEPS FEM is activated (about 800 uv) before the moment of hitting. It is interesting to note that both the RECTUS FEM and the BICEPS FEM muscles were activated after the movement of the fencer's weapon (“fencer marker”). At this point, the Fz force of the front leg disappears because the front leg moves away from the ground force reaction platform before the fencer's weapon movement. As shown in Figure 2, the fencer's front leg limb, while still in the preparatory period of the execution of the lunge, anticipatorily presses on the plate, generating a significant vertical force.

Figure 1 shows that the muscle that initiates the movement is the LAT TRICEPS, which acts as an extensor early in the latency phase. In addition, the EXT CARP RAD and BICEPS BRACHII muscles are activated later, but still in the latency phase, before the “fencer marker”. As for the FLEX CARP and BICEPS BR U muscles, there is an anticipatory activation even in the preparatory period of the execution of the fencing lunge, but below the accepted “muscle onset” threshold.

The interpretation of Figures 2, 3 should be consistent as they illustrate the coordinated action of the fencer's front and rear leg muscles during the execution of a lunge. A significant anticipatory tendency can be seen in the activation of the LAT GAS and MED. GAS muscles of the rear leg. At the same time, a vertical force effect (Fz rear) is observed in synergy with the activation of the said calf muscles.

A different activity is observed in the front leg. The RECTUS FEM and BICEPS FEM muscles are activated slightly earlier than the defined threshold for the vertical force of the rear leg, which disappears in the initial phase of the lunge when the front leg lifts off the surface of the GRF platform. In the case of Fz rear, a significant ground pressure of about 200 N is already present in the preparatory period of the lunge execution.

As shown in Table 1, the RT and MT phases of the sensorimotor responses are significantly correlated with the activation of the BICEPS BRACHII, LAT TRICEPS, EXTCARP RAD, BICEPS FEMORIS, and MED GAS muscles. Similarly, the vertical forces (Fz forward, Fz rear) show a correlation with the MT. This means that the activity of these muscles in the RT phase also has a significant effect on the speed of movement, i.e., the final efficiency of the fencing lunge.

**Table 1:** The relationships between reaction times (RT) and movement times (MT) of the fencers’ muscles and the vertical forces of the front leg (Fz forward) and the rear leg (Fz rear).

Muscle type	RT	MT
BICEPS BRACHII	0.77*	0.55
LAT RTICEPS	0.89**	0.76*
FLEX CARPI U	0.53	0.26
EXT CARP RAD	0.86*	0.72
RECTUS FEMORIS	0.41	0.40
BICEPS FEMORIS	0.78*	0.67
LAT GAS	0.71*	0.58
MED. GAS	−0.54	−0.22
FZ FORWARD	0.79*	0.60
FZ REAR	0.82*	0.62

* *p* < .05.

** *p* < .01.

*** *p* < .001.

The vertical force of the front leg is not included in Table 2 because the lifted foot loses contact with the GFR platform before the “fencer marker”. The movement time (MT) shows a statistically significant correlation with the maximum EMG value of the LAT GAS. This means that this key muscle of the rear leg decisively influences the MT of the fencing lunge.

**Table 2:** Correlations between RT and MT and maximal EMG values and vertical force of the rear leg in the studied time intervals of sensorimotor responses.

Muscle type	RT	MT
EMG BICEPS BR	0.39	0.44
EMG LAT TRICEPS	0.40	0.47
EMG FLEX CARPI U	0.25	0.34
EMG EXT CARP RAD	0.45	0.47
EMG RECTUS FEM	0.42	0.31
EMG BICEPS FEM	0.57	0.40
EMG LAT GAS	0.74	0.81*
EMG MED. GAS	0.44	0.49
FZ REAR	−0.40	−0.47

* *p* < .05.

** *p* < .01.

*** *p* < .001.

Table 3 shows strong correlations between the EMG activity of the LAT GAS and MED GAS muscles. The figures in brackets show the delay resulting from the onset of the vertical force recording for both gastrocnemius muscles. There was a significant trend of anticipatory EMG activity in relation to vertical force in MED GAS. In contrast, there was a slight anticipatory activity of the vertical force of the rear leg (Fz rear) in relation to the LAT GAS in fencers 3 and 6.

**Table 3:** EMG correlations between LAT GAS and MED GAS muscles. The delays [in ms] of the vertical force relative to the onset of the EMG activity of the studied muscles are given in parentheses.

Fencers	LAT GAS	MED GAS
Fencer 1	0.74 (0)	0.66 (0.013)
Fencer 2	0.86 (0.017)	0.87 (0.025)
Fencer 3	0.75 (−0.002)	0.67 (0.022)
Fencer 4	0.85 (0.022)	0.81 (0.030)
Fencer 5	0.85 (0.031)	0.79 (0)
Fencer 6	0.61 (−0.013)	0.74 (0.011)

All correlations were statistically significant (*p* < .05).

## Discussion

The lunge, as a fundamental technical action in fencing, has been the subject of numerous research investigations from a biomechanical and kinematic point of view (15, 16). It is an offensive action that involves a coordinated interaction of the lower and upper limbs, with the muscles of the rear leg and the attacking arm playing a key role (17, 18). As shown in the present study in the structure of activation of individual muscles, the action is initiated by the gastrocnemius muscles, then the arm extensors, followed by the forearm muscles and the biceps. The whole activation is completed by the extensors and flexors of the front leg (rectus femoris and biceps femoris).

An important novelty of the present study is the demonstration of the sequence of muscular activity in the latency phase, understood as the decisional temporal space, which can be considered equivalent to the reaction time (RT), i.e., the interval between the “coach marker” and the “fencer marker”. In this phase, the reproduction of an adequate motor program and its executive programming take place. There is no doubt that this mechanism is characterized by anticipatory actions. It was observed that immediately after the first movement of the coach, but before the visible movement of the fencer, the gastrocnemius muscles (LAT GAS and MED GAS) are the first to be activated, about 250 ms in advance. Next, the activation of the LAT TRICEPS of the sword arm is observed with a delay of about 200 ms, anticipating the onset of the BICEPS FEM and RECTUS FEM tensions in the front leg. The whole sequence is completed with the activation of the BICEPS BR and FLEX CARP U muscles of the attacking arm after the “fencer marker”, i.e., at the beginning of the movement time phase of the lunge.

Another novel contribution of the study was the use of ground force reaction platforms that were temporally integrated with the EMG and motion capture systems. The analysis of the EMG and vertical force waveforms of the FZ rear (rear leg) and FZ forward (front leg) yielded interesting results, particularly in the context of identifying anticipatory processes. Interesting data is shown in Figure 3, where the EMG curves of the gastrocnemius calf muscles are compared with the GRF curve. In the heuristic analysis, it is possible to notice the similarity of their curves. Already in the RT phase, about 100 ms before the “coach marker”, there is a significant increase of the vertical force to about 600 N and of the EMG value to 80 ms. In the movement phase, a peak of Fz rear activity to nearly 800 N and MED GAS to nearly 300 uV and LAT GAS to 250 uV is marked. As shown in Figure 2, no synergy of EMG and GRF curves is observed for the front leg. Already in the RT phase before the “fencer marker”, the Fz curve (foot lifting of the platform) disappears. On the other hand, during the movement time, the RECTUS FEM is the key muscle activated, reaching its maximum at almost 500 uV.

While emphasizing the relationship between EMG and GRF, it is important to note the interesting correlations in the period preceding the RT and MT intervals of the sensorimotor response, the so-called preparatory period. It is extremely rare for researchers to include this topic in their studies. A “smoothed” EMG signal was found by analyzing its low activity (below the accepted threshold) at the level of a few tens of microvolts [uV] just before the “ coach marker” and a reasonably firm pressure of the vertical force of the rear leg on the platform at about 200 N. On the other hand, quite unexpectedly, high values of the vertical force of the front leg were recorded during the preparatory period, at a little more than 400 N. The EMG RECTUS FEM force was 50–70 uV and the BICEPS FEM force was 20–30 uV. This phenomenon can be interpreted as an indication that during the preparatory period, the fencer, while concentrating before executing the lunge, decisively shifted her center of gravity forward, pressing her front foot against the GRF platform in anticipation. In the case of the key muscles (LAT GAS and MED GAS), a tendency of early activation of the EMG before the GRF by about 15–30 ms was recorded in six fencers studied. The opposite phenomenon was observed in two fencers, only for the LAT GAS muscle (Table 3).

The temporal aspects of the fencing lunge are worth reporting (Tables 1, 2). Of the eight muscles studied, most showed a significant influence of the RT on the MT (19).

The study proves the significant influence of the activity of the rear leg on the effectiveness of the fencing lunge, as indicated by the EMG and Fz rear force measurements. The study of the relationships between the EMG and the vertical force of the rear leg and RT and MT was justified. Only the EMG of the MAX LAT GAS muscle was shown to have a significant effect on MT (0.81).

In conclusion, the main objective of the study was to identify the factors behind the phenomenon of anticipation in combat sports. Anticipatory processes occur in the interval between the appearance of the stimulus (a visual signal) and the start of the action by the fencer, which initiates the movement phase until the lunge at the coach's torso is completed. The study showed that the anticipation mechanism affects both reaction time (RT) and the preparatory period. Thus, the phenomenon of anticipation is related to the identification, selection and programming of sensorimotor responses, as well as to the preparation of a motor activity based on previous experience and the activation of motor imagery. Both phases (RT and MT) lead to an effective execution of the technical action (lunge), in particular by reducing the time of movement execution (20).

The present study implies specific guidelines for mental and perceptual training. The former involves improving mental processes: concentration, focus and divisibility of attention, as well as reproducing correct movement patterns (21). The latter involves perfecting simple and complex reactions in conjunction with spatiotemporal anticipation. Perceptual anticipation is an important factor in reducing information processing time in the response selection phase. Studies of taekwondo and karate competitors, as well as fencers, have shown that properly conducted perceptual training affects the sensory system, allowing rapid input to neural representations and, through motor neurons, to effectors. Anticipatory information provides a pathway that paves the way for faster and more accurate selection of sensorimotor responses (7, 8). This phenomenon clearly occurs in expert fencers and, to a lesser extent, in novices.

The improvement of athletes' perceptual skills must consist of:

- developing the ability to recall and reproduce discipline-specific technical and tactical patterns;

- developing the ability to expand visual perception in response to signals related to the athlete's postural orientation;

- using strategies of divisibility and selectivity of perception (narrow and wide vision);

- and, from the point of view of motor teaching methodology, using contextual interference strategies, i.e., refining motor patterns under changing conditions. Distributed methods actively involve the trainee in the learning process by constantly changing the stimuli. They force active thinking and constant checking and comparison with previously acquired skills.

Taking a practical point of view, the results of the study confirmed the empirical data that experienced fencers already react to initial unconcious stimuli, while novices only to obvious signals. The above observations are very useful in the process of teaching and improving basic fencing techniques. Coaches should pay attention to the elements of anticipation when simulating exercises and technical-tactical solutions during individual lessons with athletes and when conducting footwork exercises. As a result, experienced fencers are characterized by lower bioelectric muscle tension and greater rationality of motor actions. This is line with the strategy of perceptual training, act with the lowest possible cost, achieving the highest results.

## Data Availability

The original contributions presented in the study are included in the article/Supplementary Material, further inquiries can be directed to the corresponding author.
